# Prevalence and Factors Associated with Contraceptive Use among HIV-Infected Women of Reproductive Age Attending Infectious Disease Clinic at Gulu Regional Referral Hospital, Northern Uganda

**DOI:** 10.1155/2018/9680514

**Published:** 2018-06-10

**Authors:** Felix Bongomin, Mercy Chelangat, Anthony Eriatu, Bruno Chan Onen, Priscilla Cheputyo, Stephen A. Godmercy, Eddymond Ekuk, Francis Idony, James Henry Obol

**Affiliations:** Department of Public Health, Faculty of Medicine, Gulu University, P. O. Box 166, Gulu, Uganda

## Abstract

**Background:**

Reproductive planning by HIV-infected women is essential, as it helps to prevent transmission of HIV to their unborn babies. Integrating contraceptive services to routine HIV care significantly increases the use of modern contraceptive methods, thus reducing vertical transmission of HIV.

**Objectives:**

To determine the prevalence and factors associated with contraceptive use among HIV-infected women attending Infectious Disease Clinic (IDC) at Gulu Regional Referral Hospital (GRRH) in Northern Uganda.

**Methodology:**

A hospital-based cross-sectional study was performed. We used simple random sampling to recruit HIV-infected women receiving routine care from IDC, GRRH, into our study. Sample size was estimated using modified Kish-Leslie formula and semistructured questionnaire was used for data collection. Data was entered into EpiData version 3.1 and analysed using Stata v11.0. We used logistic regression model to assess the associations and any factor with p≤0.05 was considered statistically significant.

**Results:**

The prevalence of contraceptive use was found to be 36% (95% CI 31 – 40%). Factors which promoted contraceptive use were as follows: being married (aOR=2.68, 95% CI 1.54-4.65, p<0.001) and monthly income of $35 -250 (aOR= 2.38, 95% CI: 1.39- 4.09, p=0.002). Factors that hindered contraceptive use were having no child (nulliparity) (aOR= 0.16; 95% CI: 0.05-0.49; p=0.002) and age range of 31-49 years (aOR= 0.53; 95% CI: 0.33 - 0.84; p=0.007).

**Conclusion:**

In this study, just over a third of sexually active HIV-infected women reported use of modern contraceptives. This is a low level of usage and, therefore, clinicians and stakeholders should sensitise HIV-infected women on the importance of contraceptive use in the fight against HIV/AIDS and encourage them to use contraceptives to avoid vertical transmission of HIV through unintended pregnancy.

## 1. Introduction

 Human Immunodeficiency Virus/Acquired Immunodeficiency Syndrome (HIV/AIDS) remains one of the most important world's public health challenges, particularly in low- and middle-income countries (LMICs). About 36.7 million people were estimated to be living with HIV worldwide at the end of 2015. Sub-Saharan Africa is the most severely affected region, with nearly 1 in every 25 adults (4.4%) living with HIV and accounting for nearly 70% of the people living with HIV (PLWHIV) worldwide [1, 2].

HIV infection primarily affects women of reproductive age worldwide, in both developed and developing countries; in fact over 50% of PLWHIV globally are women [1]. With the advent of effective highly active antiretroviral therapy (HAART), HIV-infected women are now living longer and healthier with the same reproductive goals as HIV-uninfected women [3]. However, HIV infection predisposes women to various health risks that could be deleterious to both the mother and the newborn [4]. For HIV-infected women, contraception and reproductive planning have become essential [5], as studies have shown that most of the pregnancies reported by HIV-infected women are unintended [6, 7]. The high level of unintended pregnancies increases the potential risk of mother-to-child transmission (MTCT) of HIV infection as well as the rate of induced abortions among these women [6, 7].

One of the strategies by the World Health Organization (WHO) to improve maternal health is the use of contraceptives to encourage child spacing and therefore reduce the risks of pregnancy-related complications [8]. Contraceptive use, most especially among HIV-infected women, would be of great importance in the control of their reproductive needs [8]. Over 90% of incident cases of paediatric HIV infections are attributed to vertical transmission [1]. The most effective way to eliminate mother-to-child transmission of HIV infection (eMTCT) is to ensure that the mother does not get infected with HIV; however, for those already infected, prevention of unintended pregnancies through use of highly effective modern contraceptive methods is a core eMTCT strategy [8]. Recent studies have shown that modern contraceptive methods are highly effective in reducing incidences of unintended pregnancies among HIV-infected women, including those concurrently using HAART [9]. Integrating contraceptive services as part of routine HIV care significantly increases use of modern contraceptive methods [10]. However, worldwide, a substantial unmet need for well tolerated and effective contraceptives remains, particularly in areas with a high HIV burden [3].

Uganda has the highest HIV prevalence among the East African countries estimated at 7.1%; this translates to nearly 1,500,000 PLWHIV [1, 11]. Moreover, 83,000 new HIV infections were reported in 2015, with 3,500 of these cases being diagnosed in children aged 0 to 14 years [11]. Nationally, about 120,000 pregnant HIV-infected women who need antiretroviral therapy (ART) for eMTCT are currently not able to access this life-saving medicine and thus are at an increased risk for vertical transmission [11]. The current limited access to effective contraceptive methods, eMTCT, and ART programs targeting HIV-infected pregnant women among other factors puts heavy burden on these women's reproductive health. This has resulted in unsafe abortions, increases paediatric HIV incidence through vertical transmission, and affects household incomes [12].

There is paucity of information related to use of contraceptive methods among HIV-infected women of reproductive age in the Northern region of Uganda. The present study aimed at determining the prevalence of contraceptive use and associated factors among HIV-infected women of reproductive age attending Infectious Disease Clinic (IDC) at Gulu Regional Referral Hospital (GRRH), which is a University Teaching Hospital in Northern Uganda.

## 2. Methods

### 2.1. Study Design and Setting

This was a hospital-based cross-sectional study recruiting consecutive HIV-infected women of reproductive age attending routine HIV care at the IDC, GRRH. The IDC was initially started in 2004 as a small clinic for voluntary counselling and testing for HIV (VCT). IDC currently offers a wide range of comprehensive HIV/AIDs care services including provision of HAART, eMTCT services, management of opportunistic infections, general medical consultations, and family planning services. In 2014, the clinic had over 4,000 registered clients of whom about 1,000 were females of reproductive age (15-49 years).

### 2.2. Study Population

We included nonpregnant HIV-infected women within the age range of 18 to 49 years who were registered and receiving HIV care and treatment at the IDC of GRRH for at least a month.

### 2.3. Sample Size Determination and Sampling Procedure

We estimated our sample size using the modified Kish-Leslie formula (1965) [13].

Sample size, N = (Z^2^PQ)/d^2^, where

  N is sample size;
  Z is 1.96 (standard normal deviation at 95% confidence interval);
  P is current contraceptive prevalence (we used 50% since no previous study was done in the clinic; 50% thus gives a modest number of participants);
  Q is 1-P, Q=1-0.5; therefore, Q = 0.5;
  d is maximum error we allowed between the estimated prevalence of the problem in the population; thus, d= 5% (95% confidence interval);
  N= (Z^2^PQ)/d^2^; N= ([1.96]^2^ x [0.5x0.5])/[0.05]^2^; →N= 384 respondents.

In addition, we assumed 10% lack of response/nonconsenters (0.1*∗*384 = 38) leading to an estimated sample size of [384+38] = 422 respondents.

We used simple random sampling method to enroll participants into the study. We extracted data on our research participants using a pretested data extraction form. We excluded women who were severely sick and those who had had hysterectomy. We then used simple random sampling technique without replacement to generate the names of women to be interviewed. The women were given numbers and the corresponding numbers were written in pieces of paper, folded, and placed in a nontransparent polyethylene bag, shuffled, and were then picked one at a time. The number picked was matched with the corresponding number to get the name of the participant to be interviewed.

### 2.4. Data Collection and Data Management

We used semistructured questionnaire to collect the required information. The questionnaire was translated to* Leb Acholi* (local language) and then back translated to English to ensure that the meanings of the questions were not lost during translation. FB and colleagues collected the data in their 4^th^ year of medical school after being trained by their supervisor (JHO). During the training, we pretested the questionnaire in two health facilities providing similar ART services to minimise errors during data collection. Close monitoring of data collection process was done to ensure that the questionnaires were filled correctly. Data was double-entered into EpiData version 3.1, backed up, edited, and cleaned by the researchers and supervisor to ensure data quality. Data was collected between January and May 2014.

#### 2.4.1. Variables

*Independent Variables. *Sociodemographic characteristics and reproductive factors.

*Dependent Variable. *Contraceptive use, defined as self-reported use of any of the approved modern method of contraception within the last 3 months (prior to the interview).

### 2.5. Data Analysis

Continuous variables were summarised using means and standard deviation (SD); meanwhile categorical data were summarised in terms of frequencies and percentages. Univariate analysis was performed for both dependent and independent variables. Chi-square test was performed to determine association between the independent variables and contraceptive use. Multivariate logistic regression analysis was used to assess for association between use of contraceptives and the independent predictors and to estimate crude and adjusted odds ratios and 95% confidence interval (CI). STATA version 11 (Stata Corp, College Station, Texas 77845 USA) for analysis and a P≤0.05 was considered statistically significant.

### 2.6. Ethical Considerations

The Institutional Review Committee (IRC) of Gulu University approved this study. Approval reference number is GU/IRC/04/03/13. Permission was also granted by the GRRH administration and the local administration of the IDC to conduct the study. Informed consent was obtained from all the study participants and their confidentiality were maintained. Being an undergraduate thesis research project, ethical clearance by the Uganda National Council of Science and technology was an exempt.

## 3. Results

### 3.1. Sociodemographic Characteristics

A total of 434 women were interviewed, with ages ranging between 18 and 49 years with a mean age of 30 years and SD of 7.4 years. The mean age was used to group respondents into two categories with 254 (59%) respondents being in the age range of 18 to 30 years (**Table 1**).

### 3.2. Contraceptive Use

Overall, 155 (36%) respondents were currently using at least one of the modern contraceptive methods (95% CI: 31–40, p<0.001). Of the 155 respondents, 76 (49%) were on implantable contraceptives, 49 (32%) on injectable methods, 24 (15%) on combined oral contraceptive (COC) pills, and 6 (4%) were using condoms (**Table 2**). None of the participants was on concurrent hormonal and barrier methods of contraception (dual contraception).

### 3.3. Reproductive Goals

Out of the 434 respondents, 380 (88%) had children of their own. Of the 297 respondents with current sexual partners, 277 (93%) had had children with them. Of the 434 respondents, 263 (61%) had the intention to have children in future, with 161 (61%) of these women wanting at least two children or more. However, 31 (12%) respondents reported no intentions of using contraceptives after attaining the desired number of children. Of the 263 respondents intending to have children in the future, 167 (60%) did not discuss with their partners issues related to childbirth. Of the 434 respondents, 299 (69%) reported that HIV infection could not stop them from having children (**Table 3**).

### 3.4. Factors Associated with Contraceptive Use

**Table 4** summarises results of the multivariate analysis. Statistically significant independent predictors of contraceptive utilization among our respondents were as follows: being married (adjusted odds ratio (aOR)=2.68, 95% CI 1.54-4.65, p<0.001), earning monthly income between $35 and 250 (aOR= 2.38, 95% CI: 1.39- 4.09, p=0.002), respondents having no child of their own (aOR= 0.16; 95% CI: 0.05-0.49; p=0.002), and those aged between 31 and 49 years (aOR= 0.53; 95% CI: 0.33 - 0.84; p=0.007).

## 4. Discussion

In the present study, the prevalence of contraceptive use was 36%. Majority of the women were using highly effective hormonal contraceptive methods. The prevalence of contraceptive use among HIV-infected women in our study was comparable to that of the general women population in Uganda (36% versus 35%) [14]. The contraceptive prevalence in our study is consistent with earlier studies reporting contraceptive use of 34% among HIV-infected women in Central Uganda [15] and a prevalence of 45% in Southwestern Uganda [16].

Interesting, more than half of the respondents had intention of bearing children in the future. Our data further suggests that women with higher monthly income ($35 or more) and married women are likely to use contraceptives. This could be because these women can afford the costs of contraceptives and are able to plan and discuss their reproductive goals with their partners. On the other hand, women aged 30 years or more and women who do not have children were less likely to use contraceptives. The driving force towards nonuse of contraceptives could be due to the desire for children in the face of an advancing age. The reproductive health implication of these findings suggests a targeted intervention such as health education and patient-centred counselling to create awareness on the benefits of contraception in the settings of HIV infection. Use of effective contraceptive methods reduces the incidence of unplanned and unintended pregnancies. Myer et al. found that the desire for future fertility was associated with the use of reversible contraceptives [17]. Increased use of effective contraception among HIV-infected women may potentially contribute to reduction of maternal mortality and morbidity in a country like Uganda where access to safe and legal abortions is restricted [18]. A recent study from Rakai district in Southern part of Uganda, a region with the highest HIV prevalence, showed that the prevalence of self-reported current hormonal contraceptive use by HIV-infected women had tripled over a 10-year period and was not different from that of HIV-uninfected women [19] and this is consistent with our findings.

Few studies from Uganda have scrutinized the factors that determine contraceptive use among HIV-infected women. Factors associated with increased contraceptive use included higher education, higher socioeconomic status, higher parity, age between 20 and 39 years, sexual frequency, desire to have fewer children, being currently married or in a relationship, and discussion of family planning with a partner and receipt of HIV results. On the other hand, symptoms suggestive of opportunistic infections, having no sexual partner in the past year, condom use, breastfeeding, and older age were reported to be negatively associated with contraceptive use [16, 19–21]. Almost all these factors are shared among HIV-infected and HIV-uninfected women.

Contrary to other studies from across Uganda and East Africa at large where reported use of condoms were as high as 80% [22], our study revealed that only 4% of the respondents were currently using condoms and none was on dual contraception. Use of condoms has been shown to be associated with low use of hormonal contraceptives [19]. Use of dual contraceptive methods is recommended for HIV-infected individuals irrespective of their partner's HIV sero-status to prevent HIV (re)infection, unintended and unplanned pregnancies, and other sexually transmitted infections [6]. A recent study from Uganda also reported a very low (6%) dual contraceptive use among HIV-infected women in rural Uganda where 44% of the respondents were using condoms as the main method of contraception [16].

The main limitation of this study lies in its cross-sectional design and the short duration the data was collected hence, we were unable to assess for adherence, failure rates, and adverse events related to the use of these methods among our participants. We heavily relied on self-reported use of family planning methods. However, the data from this study benefits from its large sample size and importantly explored sociodemographic and reproductive factors as core determinants of contraceptive utilization among HIV-infected women. Despite few other studies in central and southwestern Uganda, this is the first study to report the use of contraceptive methods among HIV-infected women of reproductive age in the Northern region of Uganda.

## 5. Conclusion and Recommendation

The prevalence of contraceptive use is low at just over one-third of the participants. Majority of the participants intend to have more children and this desire could be driving them not to use contraceptives. Also, women who were 31+ years were less likely to use contraceptives. Therefore, clinicians and stakeholders should sensitise HIV-infected women about the benefits of using contraceptives and encourage the use.

## Figures and Tables

**Table 1:** Sociodemographic characteristics of the respondents N=434.

**Variable**	**Frequency (n)**	**Percent (**%**)**
**Age range (18 – 49yers). Mean 30 years; SD 7.4years**		

**Age groups, n=434**		

18-30	254	59

31-49	180	41

**Education, n=434**		

No formal education	052	12

Had formal education	382	88

**Level of education, n=382**		

Primary	258	68

Secondary	117	31

Diploma	005	01

Graduate	002	01

**Marital status, n=434**		

Single	042	10

Married	276	63

Separated	068	16

Widowed	048	11

**Type of Marriage (if married), n=276 **		

Monogamous	209	76

Polygamous	067	24

**Order of Marriage (if polygamous), n=67**		

First wife	027	40

Second wife	038	57

Third wife	001	1

Fourth wife	001	1

**Religion, n=434**		

Catholic	306	71

Protestants	110	25

Muslim	016	04

Seventh Day Adventist	002	00

**Occupation, n=434**		

Peasant Farmers	235	54

Business	175	40

Civil Servants	024	06

**Monthly income, n=434**		

$0-34	258	59

$35-250	176	41

**Table 2:** Contraceptive utilization, socioeconomic, and healthcare factors.

**Variables**	**Number**	**Percent**
**Prevalence of contraceptive use 36**%**, 95**%** CI 31 – 40**%		

**Self-reported current use of contraceptive, n=434**		

Yes	155	36

No	279	64

**Family planning methods being used, n=155**		

Implants	076	49

Pills	024	15

Depo-Provera	049	32

Condoms	006	04

**Source of contraceptive, n=155**		

Government health centre	135	87

Family planning clinic (IDC)	010	06

Private clinics	003	02

Outreach	001	01

Others	006	04

**Numbers of month of uninterrupted use of contraceptive method currently used, n=155**		

1 - 16 months	102	66

17 - 84 months	053	34

**Were you told of the side effects of the contraceptive method?, n=155**		

Yes	146	94

No	009	06

**Where you advised on what to do in case of side effect?, n=155**		

Yes	144	93

No	011	07

**Were you educated on available alternative contraceptive methods?, n=155**		

Yes	144	93

No	011	07

**Cost of contraceptive, n=155**		

Free	144	93

Up to $2	011	07

**Did you discuss with your partner on the method to use?, n=155**		

Yes	124	80

No	031	20

**Do you have access to contraceptives whenever you need them?, n=155**		

Yes	151	97

No	004	03

**Receiving counselling on family planning at IDC, n=155**		

No	012	03

Yes	360	83

Sometimes	062	14

**Table 3:** Reproductive goals of the respondents.

**Variable**	**Frequency (n)**	**Percent (**%**)**
**Do you have any child of your own?, n=434**		

Yes	380	88

No	054	12

**Number of children, n=380**		

1 - 3 Children	285	75

4 - 7 children	095	25

**Do you have a child or children with current sexual partner?, n=297**		

Yes	277	93

No	020	07

**Number of children with current sexual partner, n=277**		

1 - 3 Children	216	78

4 - 7 children	061	22

**Intending to have children in the future, n=434**		

Yes	263	61

No	171	39

**Number of children intending to have in the future, n=263**		

1 Child	102	39

2 Children	122	46

3 Children	027	10

4 Children	011	04

5 Children	001	00

**After attaining required number of children, do you intent to use family planning to prevent pregnancies?, n=263**		

Yes	232	88

No	031	12

**Discussing with partner issue of child birth, n=277**		

No	167	60

Always	097	35

Sometime	013	05

**Can your HIV status stop you from getting a baby?, n=434**		

Yes	135	31

No	299	69

**Table 4:** Crude and adjusted odds ratios for factors associated with contraceptive utilization among HIV infected women of reproductive attending Infectious Disease Clinic, Gulu Regional Referral Hospital.

**Variables**	**Freq (n)**	**Contraceptive use**		**Bivariate analysis**			**Multivariate analysis**		
		**Yes (**%**)**	**No (**%**)**	**cOR**	**95**%** CI**	**P-value**	**aOR**	**95**%** CI**	**P-value**

Age groups									

18 - 30	254	105 (68)	149 (53)	1			1		

31 - 49	180	50 (32)	130 (47%)	0.55	0.36 - 0.82	**0.004**	0.53	0.33 - 0.84	**0.007**

**Marital status**									

Single/separated/widowed	158	27 (17)	131 (47)	1			1		

Married	276	128 (83)	148 (58)	4.2	2.60 - 6.76	**<0.001**	2.68	1.54 - 4.65	**<0.001**

**Educational level**									

No formal education	52	14 (9)	38 (14)	1			1		

Primary	258	85 (55)	173 (62)	1.33	0.69 - 2.59	0.397	0.91	0.43 - 1.90	0.797

Secondary	117	55 (35)	62 (22)	2.41	1.18 - 4.91	**0.016**	1.08	0.47 - 2.44	0.862

Post Secondary	7	1 (1)	6 (2)	0.45	0.05 - 4.10	0.481	0.12	0.01 - 2.90	0.192

**Religion**									

Catholic	306	100 (64)	206 (74)	1			1		

Protestants	110	45 (29)	65 (23)	1.43	0.91 - 2.23	0.121	1.36	0.80 - 2.32	0.259

Moslems	16	9 (6)	7 (3)	2.65	0.96 - 7.32	0.06	2.54	0.76 - 8.42	0.128

Others	2	1 (1)	1 (0)	2.06	0.13 - 33.27	0.611	0.64	0.06 - 7.56	0.726

**Occupation**									

Peasant Farmer	149	37 (24)	112 (40)	1			1		

Civil servant	24	12 (8)	12 (4)	3.03	1.25 - 7.31	**0.014**	2.52	0.67 - 9.49	0.173

Business	175	84 (54)	91 (33)	2.79	1.74 - 4.50	**<0.001**	1.57	0.88 - 2.82	0.126

Unemployed	78	18 (12)	60 (22)	0.91	0.48 - 1.73	0.769	1.38	0.65 - 2.94	0.402

Others	8	4 (2)	4 (1)	3.03	0.72 - 12.71	0.13	3.46	0.69 - 17.38	0.132

**Have any child of your own**									

Yes	380	151 (97)	229 (82)				1		

No	54	4 (3)	50 (18)	0.12	0.04 - 0.34	**<0.001**	0.16	0.05 - 0.49	**0.002**

**Monthly income**									

$0-34	258	64 (41)	194 (70)				1		

$35-250	176	91 (59)	85 (30)	3.25	2.16 - 4.88	**<0.001**	2.38	1.39 - 4.09	**0.002**

cOR: crude odds ratio; aOR: adjusted odds ratio.
